# WaveletHSI: Direct HSI Classification from Compressed Wavelet Coefficients via Sub-Band Feature Extraction and Fusion

**DOI:** 10.3390/jimaging11120441

**Published:** 2025-12-10

**Authors:** Xin Li, Baile Sun

**Affiliations:** College of Computer Science and Technology, China University of Petroleum (East China), Qingdao 266580, China; lix@upc.edu.cn

**Keywords:** hyperspectral image classification, compressed-domain computation, frequency domain learning, feature alignment, cross-attention mechanism

## Abstract

A major computational bottleneck in classifying large-scale hyperspectral images (HSI) is the mandatory data decompression prior to processing. Compressed-domain computing offers a solution by enabling deep learning on partially compressed data. However, existing compressed-domain methods are predominantly tailored for the Discrete Cosine Transform (DCT) used in natural images, while HSIs are typically compressed using the Discrete Wavelet Transform (DWT). The fundamental structural mismatch between the block-based DCT and the hierarchical DWT sub-bands presents two core challenges: how to extract features from multiple wavelet sub-bands, and how to fuse these features effectively? To address these issues, we propose a novel framework that extracts and fuses features from different DWT sub-bands directly. We design a multi-branch feature extractor with sub-band feature alignment loss that processes functionally different sub-bands in parallel, preserving the independence of each frequency feature. We then employ a sub-band cross-attention mechanism that inverts the typical attention paradigm by using the sparse, high-frequency detail sub-bands as queries to adaptively select and enhance salient features from the dense, information-rich low-frequency sub-bands. This enables a targeted fusion of global context and fine-grained structural information without data reconstruction. Experiments on three benchmark datasets demonstrate that our method achieves classification accuracy comparable to state-of-the-art spatial-domain approaches while eliminating at least 56% of the decompression overhead.

## 1. Introduction

**Figure 1:**
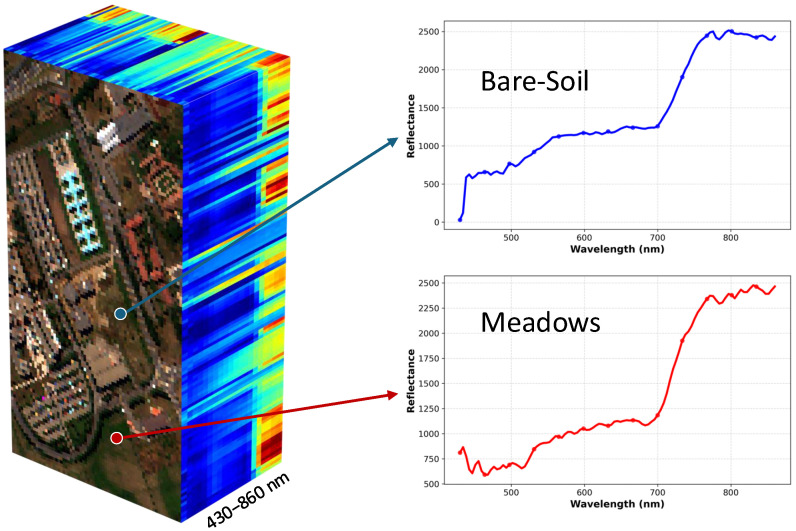
Visualization of the Pavia University HSI. The left panel depicts the three-dimensional data cube, and the right panel displays the continuous spectral signatures extracted from distinct pixels, where the blue and red curves represent the Bare-Soil and Meadows classes, respectively.

Hyperspectral imaging (HSI) acquires information across a wide range of the electromagnetic spectrum, extending well beyond the visible light captured by standard RGB cameras. Unlike conventional imagery, HSI captures hundreds of contiguous spectral bands with high spectral resolution, typically ranging from 400 to 2500 nm per band. This fine-grained spectral sampling enables the precise identification of materials and surface characteristics by analyzing their unique spectral signatures. As illustrated in Figure 1, the two classes (Bare-Soil and Meadows) exhibit distinct characteristic differences in the spectral dimension. For this reason, HSI plays a pivotal role in fields such as precision agriculture, environmental monitoring, and mineral exploration.

Despite its advantages, the high spectral resolution of HSI results in substantial data volumes, necessitating efficient compression for transmission and storage. Currently, the vast majority of remote sensing satellites employ Discrete Wavelet Transform (DWT)-based compression standards to overcome bandwidth limitations. For instance, satellites such as Yaogan 6, Ziyuan 3, and Mapping Satellite-1 utilize the Joint Photographic Experts Group 2000 (JPEG2000) standard [1,2,3]. However, the subsequent decompression process creates a computational bottleneck for large-scale data analysis. Assuming that decompressing a single 200-band HSI takes approximately 10 s, processing 10,000 such samples would require nearly 28 h. This latency underscores the necessity for compressed-domain computing methods capable of processing data directly in its compressed form without full reconstruction.

Compressed-domain computing has emerged as a potential solution to this efficiency challenge. While this paradigm has been explored in natural image processing, existing approaches primarily focus on the JPEG standard, which is based on the Discrete Cosine Transform (DCT) [4,5,6]. Unfortunately, these methods are not directly transferable to HSI due to the structural disparity between transform domains. The DCT domain utilizes an 8×8 block-based structure with intermixed spatial and frequency components, whereas the DWT domain features a hierarchical, multi-resolution sub-band structure. Existing DCT-based architectures, which often apply uniform projections to blocks, fail to accommodate the frequency independence and multi-scale nature inherent in the DWT structure.

Consequently, a fundamental research objective arises: How can we formulate a unified framework for HSI classification that operates efficiently and effectively within the DWT compressed domain?

Establishing such a framework presents two primary challenges derived from the specific data structure of DWT sub-bands. First, unlike the single tensor input in pixel-domain networks, DWT decomposes images into multiple sub-bands (e.g., LL, LH, HL, HH) with varying resolutions, necessitating a multi-branch architecture to extract features in parallel. Second, the semantic information is distributed across different frequency bands; thus, effectively fusing high-frequency details with low-frequency approximations is critical to prevent information loss during the classification process.

To address these challenges, we propose a novel compressed-domain classification framework named WaveHSI. To handle the multi-branch input, we design a Multi-Branch Multi-Scale Spatial-Spectral Feature Extraction Module ($M^{2}S^{2}$-FEM) with a Sub-band Feature Alignment (SFA) loss that processes wavelet sub-bands directly. Furthermore, to resolve the feature fusion challenge, we introduce a Sub-band Cross-attention Feature Fusion Module (SCA-FFM), which leverages high-frequency components to refine the semantic representation of low-frequency features.

The main contributions of this paper are summarized as follows:

- We explore a novel feature learning paradigm for the hierarchical DWT structure. By proposing the $M^{2}S^{2}$-FEM with a specialized alignment loss, we demonstrate that robust spatial–spectral representations can be learned directly from decoupled wavelet sub-bands, bypassing the inverse wavelet transform.
- We investigate the interaction mechanism between varying frequency layers. To this end, we design the SCA-FFM, which reveals that leveraging high-frequency details to guide the refinement of low-frequency features via cross-attention effectively mitigates the information loss in deep networks.
- We present a high-efficiency framework that achieves a superior trade-off between accuracy and speed. Comprehensive experiments on three benchmark datasets validate that our method reduces computational overhead, offering a promising solution for time-critical remote sensing applications.

## 2. Background and Related Works

### 2.1. Wavelet Compression Method

**Figure 2:**
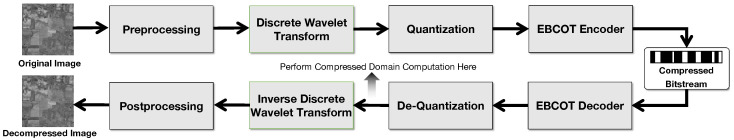
JPEG2000 compression and decompression process.

Many HSI compression algorithms utilize DWT, with JPEG2000 being the most prominent example. Based on JPEG2000, this paper explores how to implement deep learning computations directly in the compressed-domain. As shown in Figure 2, the JPEG2000 compression process comprises the following steps: (1) Preprocessing: including image tiling, DC shifting, and component transformation, which provide a more suitable data representation for subsequent wavelet transform and encoding processes. (2) DWT: This is the core step of the JPEG2000 algorithm, decomposing the image into four frequency sub-bands that capture both high-frequency and low-frequency information of the image. (3) Quantization of wavelet coefficients: This lossy step discards certain data to reduce the overall file size. (4) Encoding of quantized wavelet coefficients using Embedded Block Coding with Optimized Truncation (EBCOT) to obtain a compressed bitstream. EBCOT is an entropy encoding technique used to further compress image data and remove redundancy.

Throughout this process, the computational complexity of the wavelet transform is O($N^{2}$), where N is the image side length [7]. This quadratic complexity causes significant computational delays for large-scale HSI, with delays increasing with the number of hyperspectral bands and wavelet decomposition levels. For large amounts of high-resolution hyperspectral data, omitting the inverse wavelet transform step can save considerable time. Beyond computational efficiency, the wavelet domain offers inherent advantages for feature representation. Distinct regions of the electromagnetic spectrum manifest unique frequency characteristics; for instance, the subtle contrast variations found in near-infrared bands correspond to specific high or low-frequency components. These features often exhibit superior discriminability in the frequency domain compared to the spatial domain, thereby validating the rationale for performing analysis directly on wavelet coefficients. Moreover, JPEG2000 provides lossless wavelet transform capabilities, allowing the transformed image to retain all information. The multi-resolution representation provided by multi-level wavelet transform enables easy access to different detail levels of the image, which are prerequisites for feasible compressed-domain computation. Therefore, following previous works [8,9,10], this paper defines the compressed-domain as the multi-level wavelet domain data obtained after EBCOT decoding and before inverse wavelet transform. We propose a method to directly input this data into neural networks without fully reconstructing the image.

### 2.2. DCT Compressed-Domain Computing

**Figure 3:**
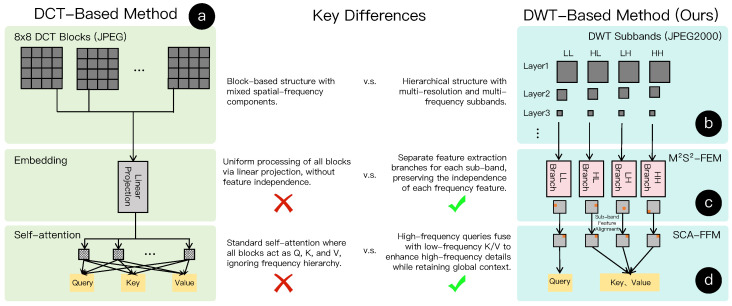
Structural comparison between DCT-based and DWT-based compressed-domain paradigms. (**a**) DCT-based methods typically process 8×8 blocks with mixed spatial-frequency components using uniform projections. (**b**) In contrast, the DWT domain exhibits a hierarchical structure with distinct frequency sub-bands, which existing block-based methods fail to address. (**c**,**d**) illustrate the proposed sub-band independent processing and feature alignment strategies.

While this paper focuses on wavelet-based compression, the paradigm of compressed-domain computing has matured significantly within the context of DCT-based JPEG images. As illustrated in Figure 3a, the standard DCT compression pipeline partitions an image into independent 8×8 blocks. Within each block, spatial and frequency information are intermixed, and existing methods typically apply uniform projections to these blocks to extract features.

Gueguen et al. [4] pioneered this direction by modifying ResNet to accept DCT coefficients directly from the decoded blocks. By bypassing the computationally expensive inverse DCT and RGB conversion steps, they achieved significant acceleration in image classification. Subsequently, Park et al. [5] identified a natural alignment between the 8×8 DCT blocks and the patch embedding mechanism of Vision Transformers. They proposed treating flattened DCT blocks as input tokens, allowing the network to learn directly from frequency representations without resizing artifacts. To further optimize the trade-off between accuracy and efficiency, Li et al. [6] introduced a learnable frequency selection mechanism (DCFormer) to filter out redundant high-frequency channels within the DCT blocks.

However, these successes in the DCT domain do not readily translate to HSI processing. As shown in the comparison between Figure 3a,b, there is a fundamental structural disparity. DCT methods rely on the independence of local blocks, whereas the DWT standard (JPEG2000) used in HSI features a global, hierarchical multi-resolution structure where frequency sub-bands are explicitly separated. This mismatch necessitates the development of specialized DWT-based frameworks, as discussed in the following section.

### 2.3. DWT Compressed-Domain Computing

Extensive explorations into the JPEG2000 compressed-domain have been conducted prior to this work. As early as 2005, Fehmi Chebil et al. [10] proposed a method for directly editing images in the JPEG2000 compressed-domain. By manipulating wavelet coefficients, they enabled common image editing operations such as brightness adjustment, contrast adjustment, image overlay, and linear filtering, thereby improving editing efficiency. When processing high-resolution images, this method enhanced editing performance by more than 10 times.

Fan Zhao et al. [11] proposed an adaptive blind watermarking algorithm based on the JPEG2000 compressed-domain. This algorithm fully utilizes the rich bitstream syntax of the JPEG2000 standard and enables copyright protection for JPEG2000 images by embedding watermarks in the compressed-domain. In 2023, Bisen et al. [12] proposed a method for directly extracting text and non-text regions within the JPEG2000 compressed-domain. This method utilizes JPEG2000 characteristics (such as resolution and bit depth) to efficiently separate text and non-text regions, and extracts layout information through the maximally stable extremal regions algorithm, thus saving computing resources and improving processing efficiency.

Li et al. [13] proposed an analysis workflow for whole slide image processing based on the compressed-domain. By utilizing the compressed-domain pyramid magnification structure and compressed-domain features obtained from the original bitstream, they achieved efficient analysis of whole slide image classification models. This method reduces computing resource consumption while maintaining model accuracy comparable to that of the original workflow.

In the remote sensing field, Akshara et al. [8] pioneered compressed-domain work on RGB remote sensing images. Their approach uses transposed convolutional layers to approximate higher-resolution wavelet sub-bands from the coarsest-resolution wavelet sub-bands, then employs multiple convolutional layers to extract features from high-resolution wavelet sub-bands, and finally uses a fully connected layer for classification. Although using a simple network structure, they demonstrated that compressed-domain computation can significantly reduce computing time and storage requirements while maintaining high classification accuracy when processing large-scale remote sensing image data.

Table 1 categorizes existing compressed-domain methods by their transform domain (DCT vs. DWT). Although these excellent methods have achieved promising performance on natural images and RGB remote sensing classification, no method for HSI classification tasks has been proposed, and this paper aims to fill this gap.

## 3. Preliminaries: Wavelet Transform Based Data Structure

To clarify the input data format for our proposed method, this section details the structure of HSI data in the wavelet domain. This structure results from the 2D DWT used in compression standards like JPEG2000, where each HSI band is recursively decomposed. The process begins by transforming the initial data (H×W×B) into a low-frequency sub-band (${\mathrm{LL}}_{1}$) and three high-frequency sub-bands (${\mathrm{HL}}_{1},{\mathrm{LH}}_{1},{\mathrm{HH}}_{1}$). The recursive relationship is defined as:

(1) $${\mathrm{LL}}_{k},\left\{{\mathrm{LH}}_{k},{\mathrm{HL}}_{k},{\mathrm{HH}}_{k}\right\}=DWT_{k}\left({\mathrm{LL}}_{k-1}\right)$$

where $DWT_{k}\left(\cdot \right)$ is the *k*-th level DWT. The dimensions of the sub-bands at level *k* are $\frac{H}{2^{k}}\times \frac{W}{2^{k}}$. We use the same notations for LL,HL,LH,HH as Akshara et al. [8] did.

For instance, performing a 3-level decomposition on a single band with initial dimensions of 610×340 produces the following: the first level generates four sub-bands (${\mathrm{LL}}_{1},{\mathrm{HL}}_{1},{\mathrm{LH}}_{1},{\mathrm{HH}}_{1}$) of size 305×170; the ${\mathrm{LL}}_{1}$ sub-band is then decomposed into four smaller sub-bands (${\mathrm{LL}}_{2},\ldots$) of size 153×85; finally, the ${\mathrm{LL}}_{2}$ sub-band is decomposed into sub-bands (${\mathrm{LL}}_{3},\ldots$) of size 77×43.

**Figure 4:**
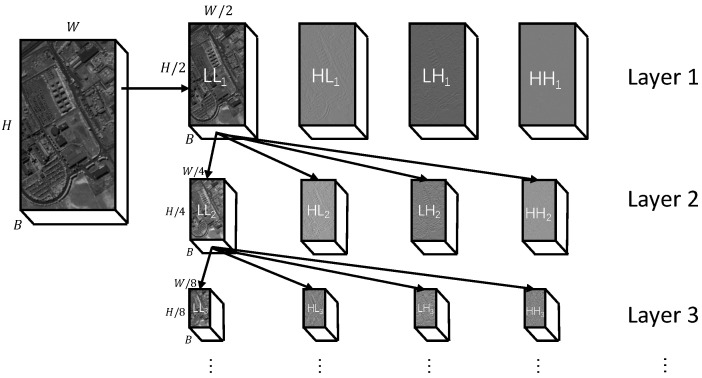
Illustration of wavelet transform based data structure.

As calculated above, a 4th level would reduce the LL band to approximately 38×21. This resolution is insufficient for the subsequent convolutional or attention-based modules to capture meaningful spatial context. Therefore, in this work, we utilize 3 levels to maintain a resolution of 77×43, achieving an optimal balance between frequency domain separation and spatial semantic preservation. The resulting data structure consists of three different scales, as summarized in Figure 4.

This wavelet domain structure provides the foundation for our method. Its multi-scale nature motivates the architecture of our M^2^S^2^-FEM module. The functional separation between the information-rich LL sub-bands and the detail-oriented HL, LH, HH sub-bands justifies a multi-branch processing strategy and the cross-attention fusion in our SCA-FFM. By operating directly on these coefficients instead of the reconstructed image, our method bypasses the computationally expensive inverse transform, enabling compressed-domain computing.

## 4. Methods

This section introduces the workflow of the WaveHSI method proposed in this paper. Section 4.1 provides an overall introduction to the entire pipeline. Section 4.2 elaborate on the specific designs of the two modules: $M^{2}S^{2}$-FEM and SCA-FFM. Section 4.3 presents the training strategy, including a sub-band random masking strategy proposed to prevent overfitting, as well as a detailed introduction to the loss functions.

### 4.1. Overview

**Figure 5:**
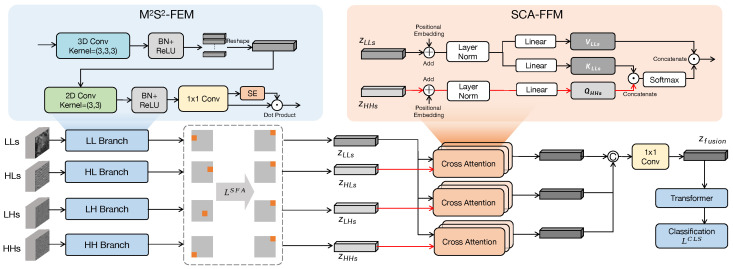
Illustration of $M^{2}S^{2}$-FEM and SCA-FFM.

After obtaining the compressed-domain data in the form described in Section 3, the WaveHSI implementation proceeds through the following four steps:

(1) Data preprocessing: The HSI classification task is pixel-level, requiring prediction results for each pixel. Since the resolution of DWT domain data cannot be spatially aligned with the original image, all sub-bands are first upsampled to the spatial size of the original data using bilinear interpolation. Then, to reduce input dimension and improve computational speed, Principal Component Analysis is applied to reduce the spectral dimension of each sub-band to $d_{\mathrm{pca}}$. To expand the contextual spatial information of input pixels, patches are extracted with each pixel as the center and *r* as the radius, using the patch instead of a single pixel as the input.
(2) Shallow feature extraction: Since LL, HL, LH, and HH represent features in different directions, each branch needs to be processed separately. As shown in Figure 5, we designed $M^{2}S^{2}$-FEM to extract shallow features from the four sub-bands. This module has four branches with the same network structure. Each branch extracts spectral features through 3D convolution and then spatial features through 2D convolution, obtaining shallow features $z_{\mathrm{LL}},z_{\mathrm{HL}},z_{\mathrm{LH}},z_{\mathrm{HH}}$ from the four sub-bands. To ensure the alignment of features across different branches, we employ SFA loss. This loss functions by using the spatial layout as an intermediary, compelling the channels in each sub-band feature to exhibit specific spatial activation patterns.
(3) Deep feature fusion: $z_{\mathrm{LL}}$ represents low-frequency features that retain most information and are of highest importance for classification, while other high-frequency features play auxiliary roles. To fully fuse the information from low-frequency and high-frequency features, we propose SCA-FFM. This module uses the features of HL, LH, and HH as queries, and the features of LL as keys and values to compute attention, obtaining high-level fused features.
(4) Classification: Following the traditional vision transformer-based classification process, we pass the fused features into the transformer encoder, obtain the classification token after a series of nonlinear transformations, and finally obtain the classification result through a linear layer.

### 4.2. Model Architecture

#### 4.2.1. Multi-Branch Multi-Scale Spatial–Spectral Feature Extraction Module

**Figure 6:**
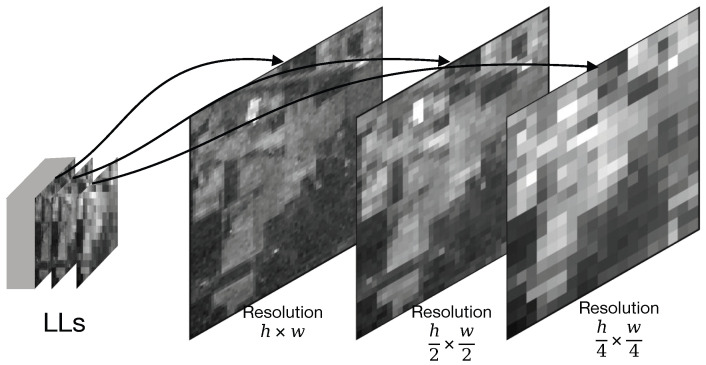
Input data of the $M^{2}S^{2}$-FEM (taking LLs of three-level wavelet decomposition as an example).

The $M^{2}S^{2}$-FEM module is designed to effectively extract multi-scale spatial–spectral features directly from distinct sub-bands generated by the DWT, addressing the challenge of capturing hierarchical context from wavelet coefficients. To achieve this, the module’s input is constructed through a specialized channel concatenation strategy. As illustrated in Figure 6, patches from different decomposition levels corresponding to the same spatial location are arranged adjacently along the channel dimension. This organization allows subsequent convolutional kernels to simultaneously process features from multiple resolutions, facilitating hierarchical correlation learning. This approach directly leverages the multi-scale outputs of the wavelet domain, unlike methods such as spatial pyramid pooling [14] that generate scale invariance through pooling operations.

Inspired by the Mixture of Experts (MoE) architecture [15,16] in the field of large language models, we employ a multi-branch architecture with four parallel streams to process this structured multi-scale input, each dedicated to a specific sub-band type (LLs, HLs, LHs, and HHs). Each branch utilizes 3D convolution to extract initial spatial–spectral features, with kernels operating across both spatial dimensions and the concatenated scale-channel dimension to capture local patterns and spectral-scale correlations. This is followed by depthwise separable convolution, which first isolates spatial pattern learning through depthwise convolution and then efficiently combines channel information via pointwise convolution, enhancing spatial texture extraction while reducing computational overhead. To further refine feature representations, a Squeeze-and-Excitation (SE) block is incorporated in each branch to adaptively recalibrate channel importance, amplifying informative features while suppressing noise. The outputs of this process are the refined feature maps for each sub-band group: $z_{\mathrm{LLs}}$, $z_{\mathrm{HLs}}$, $z_{\mathrm{LHs}}$, and $z_{\mathrm{HHs}}$.

The LLs, HLs, LHs, and HHs branches extract features in parallel. However, this independent extraction design lacks an explicit constraint to ensure the output features from the four branches are spatially aligned with each other. To reinforce the spatial consistency and to enhance the model’s representation capability for subtle local objects, we introduce SFA Loss. Inspired by Liu et al. [17], the core idea of it is to leverage a spatial consistency prior by explicitly binding the feature channels of each branch to fixed spatial regions. By forcing all branches to adhere to the same spatial alignment rule, we indirectly achieve alignment across the different branches. The detailed calculation process is presented in Section 4.3.2.

#### 4.2.2. Sub-Band Cross-Attention Feature Fusion Module

Compared with existing feature fusion methods [18,19], our main challenge lies in performing feature fusion across multiple wavelet sub-bands ($z_{\mathrm{LLs}},z_{\mathrm{HLs}},z_{\mathrm{LHs}},z_{\mathrm{HHs}}$) after feature extraction by the $M^{2}S^{2}$-FEM. Simple concatenation can be suboptimal as it treats low-frequency and high-frequency sub-bands equally, potentially combining noise with signal. Similar to many multimodal studies focusing on cross-attention-based feature fusion [20,21], we propose SCA-FFM to address this issue. This module uses a cross-attention mechanism for adaptive fusion where high-frequency features query low-frequency features to integrate details with the primary signal.

Before the cross-attention step, each sub-band feature set passes through a pooling layer that applies adaptive average pooling and max pooling in parallel. This extracts both global and local context from the feature maps. Average pooling provides feature summaries, while max pooling extracts features with the highest activation. Using both provides the attention mechanism with information about general statistics and prominent features.

The central component of SCA-FFM is its cross-attention mechanism, where features from high-frequency sub-bands ($z_{\mathrm{HLs}},z_{\mathrm{LHs}},z_{\mathrm{HHs}}$) serve as queries, while features from the low-frequency sub-band ($z_{\mathrm{LLs}}$) serve as both keys and values. This configuration allows high-frequency features to attend to low-frequency features, weighting low-frequency information based on its relevance to high-frequency details. The computation is performed using a multi-head attention structure to capture relationships in different representation subspaces.

The attention mechanism output is then processed by 1 × 1 convolution to project features into a new dimension, followed by a transformer encoding layer to further refine the features, producing the module’s final output. The goal of this module is to generate a unified feature representation by combining information from low- and high-frequency sub-bands through a data-driven attention process.

### 4.3. Training Strategy

#### 4.3.1. Sub-Band Random Masking Strategy

To force the network to learn cross-modal complementary feature representations and reduce dependence on a single sub-band, we designed a sub-band random masking strategy during the training phase. Specifically, after convolution and before the SE attention, random masking *M* is applied to sub-band features (LLs/HLs/LHs/HHs):

(2) $$z_{\mathrm{LLs}/\mathrm{HLs}/\mathrm{LHs}/\mathrm{HHs}}^{\mathrm{masked}}=z_{\mathrm{LLs}/\mathrm{HLs}/\mathrm{LHs}/\mathrm{HHs}}⊙M,\;$$

where ⊙ denotes element-wise multiplication. And the mask matrix *M* satisfies:

(3) $$M_{i,j,k,l}=\left\{\begin{array}{cc} 0 & \mathrm{with}\;\mathrm{probability}\;p\\ 1 & \mathrm{with}\;\mathrm{probability}\;1-p \end{array}\right.\;$$

where *p* is a hyperparameter. The discussion on the value of *p* is presented in the Section 6.1.4.

The sub-band random masking strategy not only enhances cross-modal compensation learning but also trains the channel attention module’s ability to filter important features, ensuring the network has sufficient generalization ability and preventing overfitting.

#### 4.3.2. Loss Function

Two loss functions are employed in the method proposed in this paper: one is the SFA loss denoted as $L^{SFA}$, and the other is the CLS loss denoted as $L^{CLS}$.

The SFA loss is applied independently to each of the four feature maps output by the M^2^S^2^-FEM module ($z_{LLs},z_{HLs},z_{LHs},z_{HHs}$). For any given branch’s output feature map $z\in R^{d\times d\times w}$, where *D* is the number of channels and h×w is the spatial resolution, our grouping strategy is as follows: we first divide the *d* feature channels into h×w groups, with each group containing c=d/(h×w) channels. We then assign a fixed, artificial “region label” $y^{k}=j$ to all channels within the *j*-th group (where j∈[0,…,h×w−1]). This label *j* corresponds to the *j*-th pixel location in the h×w spatial grid. We enforce this alignment using a cross-entropy loss function, formulated as

(4) $$L^{SFA}=\frac{1}{d}\sum_{k=1}^{d}-y^{k}\log\left(flatten\left(z^{k}\right)\right),\;$$

where $z^{k}\in R^{h\times w}$ is the feature map of the *k*-th channel. $flatten(z^{k})$ represents the flattened feature map, which can be converted into an h×w-dimensional probability vector (optionally via Softmax) representing the channel’s activation distribution across all spatial locations. $y^{k}$ is the ground-truth region label (*j*) assigned to the *k*-th channel, represented as an h×w one-hot vector with a `1’ only at the *j*-th position. This loss function compels the activation energy of the *k*-th channel (belonging to the *j*-th group) to concentrate primarily on the *j*-th spatial pixel location. By applying this $L^{SFA}$ simultaneously to all four sub-band branches, we ensure that the low-frequency global features ($z_{LLs}$) and the high-frequency detail features ($z_{HLs},z_{LHs},z_{HHs}$) are aligned before entering the SCA-FFM.

In the classification head, we employ the standard Cross-Entropy loss $L^{CLS}$, which measures the discrepancy between the model’s predicted probability and the true label distribution:

(5) $$L^{CLS}=-\sum_{i=1}^{bs}\sum_{j=1}^{C}y_{ij}\log\left({\hat{y}}_{i,j}\right),$$

where bs is the batch size, *C* is the number of classes, $y_{ij}$ is the true label for the *i*-th sample belonging to the *j*-th class, and ${\hat{y}}_{i,j}$ is the corresponding predicted probability.

Our final objective function, $L^{Total}$, is a weighted sum of $L^{CLS}$ and SFA losses applied to each of the four sub-band branches from the M^2^S^2^-FEM:

(6) $$L^{Total}=L^{CLS}+\lambda \sum_{s\in \{LLs,HLs,LHs,HHs\}}L_{s}^{SFA}\;$$

where λ is a hyperparameter that balances the contribution of the classification task and the auxiliary feature alignment task. During training, we select the model that achieves the smallest total loss as the best model.

## 5. Experimental Setup

### 5.1. Datasets

- Indian Pines: It is acquired by NASA’s airborne visible/infrared imaging spectrometer sensor over northwestern Indiana, contains 145×145 pixel imagery with 200 spectral bands (400–2500 nm) after preprocessing. The scene was captured in June, predominantly features farmland and woodlands with 16 land-cover categories. Ground truth annotations are available through Purdue University’s MultiSpec platform.
- Pavia University: It comprises a HSI captured by reflective optics system imaging spectrometer sensor over Pavia, Italy. It consists of 610×340 pixels with 225 spectral bands (430–860 nm), each with a spatial resolution of 1.3 m. The image is divided into 9 classes: asphalt, meadows, gravel, trees, painted metal sheets, bare soil, bitumen, self-blocking bricks, and shadows.
- Kennedy Space Center: It is acquired by NASA’s sensor over Kennedy Space Center, Florida, on 23 March 1996, with 18 m spatial resolution from 20 km altitude, featuring 224 spectral bands (10 nm width, 400–2500 nm center wavelengths) and 176 retained after excluding water absorption and low signal-to-noise ratio bands. The dataset encompasses 13 land cover classes, developed to reflect discernible functional types, with classification challenges due to similar spectral signatures among certain vegetation.

As shown in Table 2, the dataset splitting ratios (training:validation:testing) were set as 10%:1%:89% for the Indian Pines and Kennedy Space Center datasets, and 5%:0.5%:94.5% for the Pavia University dataset, respectively. A random sampling strategy was adopted to select samples for model training, validation, and testing, and this ratio setting was consistent with the work of Li et al. [22] to ensure the comparability of experimental results.

### 5.2. Implementation Details

All experiments were conducted on a workstation equipped with an NVIDIA GeForce RTX 4090D GPU, implemented using the PyTorch framework (version 2.5.1). We set the radius *r* of the patches to 6, resulting in a patch size of 13, and this configuration has been validated as effective in GraphGST [23]. We utilized the Adam optimizer with a fixed learning rate of $1\times 10^{-3}$. The batch size was set to 64, and each model was trained for 100 epochs. To mitigate the impact of randomness on the comparative experiments in Section 6.2, we performed 10 independent runs for each method on every dataset. The experimental results are reported as the “mean ± standard deviation”.

### 5.3. Evaluation Metrics

In this paper, the classification performance is evaluated using three widely adopted accuracy metrics: Overall Accuracy (OA), which represents the proportion of correctly classified pixels among all pixels; Average Accuracy (AA), which is the average of the classification accuracies for each class; and Kappa Coefficient (KA), which accounts for the agreement between the classified results and the reference data while considering the possibility of random chance. Additionally, the decode time is employed to measure the efficiency of the inverse discrete wavelet transform decode process. The inference time refers to the time taken for model inference.

### 5.4. Baselines

We selected 12 methods spanning from 2004 to 2024 as the baseline. They are categorized as follows: Traditional Machine Learning Methods: Support Vector Machine (SVM) (2004) [24], Random Forest (RF) (2005) [25]. Deep Learning-Based Methods: ContextNet (2017) [26], RSSAN (2020) [27], SSTN (2022) [28], SSAN (2020) [29], SSSAN (2022) [30], SSAtt (2021) [31], A2S2KResNet (2021) [32], CVSSN (2023) [22], IGroupSS-Mamba (2024) [33], GraphGST (2024) [23].

## 6. Results and Analysis

### 6.1. Ablation Studies

#### 6.1.1. Layers in Wavelet Transform

In the JPEG2000 algorithm, the number of wavelet decomposition layers in the compression process is a hyperparameter, which is typically set to 3–6 layers. As introduced in Section 3, when the number of wavelet decomposition layers exceeds 3, the resolution becomes too small to extract effective semantic information. Additionally, to simplify the computation, we set the number of wavelet decomposition layers to 3 and investigate the experimental effects of decomposition at different levels. L1, L2, L3, and FULL represent the first layer, second layer, third layer, and full decompression, respectively.

As shown in Table 3, the performance of using L2 or L1 alone is better than that of using L3 alone. This is because L3, as the bottommost layer of wavelet decomposition, has its feature map size reduced significantly after multiple downsampling steps, resulting in the loss of a large amount of detailed information (such as key discriminative features like edges and textures) during compression, which limits the feature expression capability. The performance of using multiple layers, such as L3+2 or L3+2+1, is better than using a single layer. The reason is that multi-layer wavelet decomposition corresponds to feature spaces of different scales: L3 retains global contour information, L2 contains medium-scale details, and L1 focuses on local fine-grained features. The fusion of multiple layers can complement the semantic information of different levels, enrich the hierarchical structure of feature representation, and thereby enhance the model’s discriminative ability for complex scenes. In general, using L3+2+1 can achieve relatively good performance. Therefore, in subsequent accuracy comparison experiments, we will adopt L3+2+1.

In terms of time reduction, the more times the inverse DWT is performed, the more decoding time is consumed. On the three datasets, compared with full decompression, using only L3 requires no decoding time. The decoding time for using only L2 and using L3+2 is reduced by 83%. The decoding time for using only L1 and using L3+2+1 is reduced by 56%. This means that our compressed-domain computing method can save at least 56% of the decompression time while achieving good accuracy.

#### 6.1.2. Sub-Band Feature Alignment

To validate the effectiveness of the proposed SFA loss and determine the optimal hyperparameter settings, we conducted two ablation studies. The impact of the balancing parameter λ in the SFA loss function is first investigated, and the results are presented in Table 4. We observe a clear trend where the classification performance initially improves as λ increases, reaches its peak, and then declines. Specifically, when λ is set to 1 × 10^−2^, the model achieves the highest scores across all three key metrics: KA (99.39%), OA (99.54%), and AA (99.07%). This indicates that a moderate weighting of the SFA loss is most beneficial for feature alignment. Values that are too small (1 × 10^−4^, 1 × 10^−3^) may lead to insufficient alignment, while values that are too large (1 × 10^−1^, 1) could potentially disrupt the primary classification task, resulting in a performance drop. Therefore, we adopt λ = 1 × 10^−2^ for all subsequent experiments.

We then performed an ablation study to directly assess the contribution of the SFA loss itself. Table 5 compares the classification performance of our model with and without the SFA loss. The results demonstrate that incorporating the SFA loss consistently enhances the model’s feature representation capabilities, leading to superior classification accuracy. This confirms that the SFA loss effectively aligns features from different sub-bands, reducing intra-class variance and enhancing inter-class discriminability, which ultimately boosts the overall classification performance.

#### 6.1.3. Number of Cross-Attention Blocks

To investigate the effectiveness of the cross-attention mechanism in feature fusion and determine the optimal configuration of its block quantity, we gradually increased the number of cross-attention blocks from 1 to 6 and applied them to different feature combinations of wavelet transform layers.

As shown in Table 6, the experimental results reveal the critical role of the number of cross-attention blocks and feature input combinations in model performance. Under both L3+2 and L3+2+1 configurations, the number of cross-attention modules exhibits a performance curve that “first increases and then decreases.” Specifically, as the number of modules increases from 1 to 5, model performance improves steadily and reaches its peak at 5 layers. When the number of modules increases to 6, all performance metrics show significant decline. This phenomenon indicates that 5 cross-attention blocks achieve the optimal balance between effectively capturing and fusing multi-scale features while avoiding model overfitting, whereas an excessive number of modules may introduce redundant parameters, thereby impairing the model’s generalization ability.

#### 6.1.4. Effectiveness of Different Sub-Band Random Masking Strategies

To verify the effectiveness of the wavelet sub-band-based random masking regularization method proposed in this paper and explore the impact of different masking strategies on model performance, we designed a series of ablation experiments. The experiments used a model without any masking strategy (no mask) as the baseline, testing different mask ratios and masking sub-bands. For mask ratios, we compared two different intensities: 10% (0.1) and 1% (0.01). For masking regions, we designed two strategies: one applies masking only to high-frequency sub-bands (HL, LH, HH) to preserve the complete structural information of the low-frequency approximation sub-band (LL); the other applies masking to all four sub-bands (LL, HL, LH, HH).

As shown in Table 7, the experimental results demonstrate the effectiveness of the random masking strategy in improving model performance. Among all experimental configurations, applying a 0.1 mask ratio to all four sub-bands (LL, HL, LH, HH) achieved optimal performance. Particularly when using L3+2+1, this strategy’s KA, OA, and AA reached 98.80%, 98.95%, and 99.09% respectively, comprehensively surpassing the baseline model without masking and all other masking strategies. Further analysis revealed that the 0.1 mask ratio significantly outperformed 0.01, as the latter failed to provide effective regularization due to insufficient perturbation. Interestingly, although preserving LL sub-band information is effective, applying appropriate masking to the LL sub-band can yield better results. This indicates that slightly perturbing low-frequency approximation information can prompt the model to rely not only on the signal’s macro structure but also to more effectively learn and fuse detailed and texture features contained in high-frequency sub-bands, thereby obtaining stronger generalization ability and robustness.

### 6.2. Comparative Analysis of Classification Accuracy

WaveHSI not only saves decompression time but also achieves excellent accuracy. As shown in Table 8, Table 9 and Table 10, the experimental results demonstrate the superior performance of our proposed method in HSI classification across multiple datasets.

For the Indian Pines dataset, our method achieves an OA of 98.60%, AA of 98.22%, and Kappa of 98.41%. In terms of statistical stability, our method yields a standard deviation of ±0.17% in OA, indicating more consistent convergence compared to baselines such as IGroupSS-Mamba (±1.14%). regarding specific classes, our method reaches 100.00% accuracy on “Corn” and performs effectively on spatially scattered categories, achieving 97.66% on “Soybean-notill” and 99.11% on “Soybean-mintill”.

On the Pavia University dataset, our method records the highest performance metrics with an OA of 99.77% and AA of 99.63%. The stability of the approach is evidenced by the minimal variance (±0.05%) in OA. Class-wise analysis shows that our method classifies “Bare-soil” with 100.00% accuracy. Furthermore, it addresses the classification challenges in the “Gravel” class, achieving 99.95% accuracy, whereas competitive models like GraphGST decline to 91.29%.

Regarding the Kennedy Space Center dataset, our method attains an OA of 98.43% and AA of 97.65%. Statistically, the proposed method demonstrates improved robustness with a standard deviation of ±0.29% in OA, contrasting with the higher fluctuation observed in CVSSN (±2.58%). While GraphGST exhibits performance degradation on specific classes such as “Slash-pine” (0.22%), our method maintains consistent accuracy across all categories, recording 99.77% on “Slash-pine” and 98.51% on “Oak/B-hammock”.

### 6.3. Efficiency Analysis

To evaluate the computational efficiency of the proposed framework, we compared the decoding time, inference time, and total processing time against two state-of-the-art baselines: IGroupSS-Mamba and GraphGST. The quantitative results on three datasets with varying spatial dimensions are summarized in Table 11.

As observed, while our method (utilizing L3+2+1) exhibits higher inference latency compared to the lightweight GraphGST model, it demonstrates a substantial advantage in decoding efficiency. On smaller datasets such as Indian Pines (145×145×200) and Pavia University (610×340×103), the total processing time is dominated by the inference stage, resulting in no significant speedup. However, the benefits of compressed-domain computing become prominent on the larger Kennedy Space Center dataset (512×614×176). In this scenario, our method achieves the lowest total time of 516.6 ms, significantly outperforming IGroupSS-Mamba (3193.5 ms) and GraphGST (1383.6 ms). This efficiency gain is primarily attributed to the elimination of the computationally expensive inverse DWT process, validating the scalability and potential of our method for large-scale hyperspectral data processing.

## 7. Discussion

### 7.1. Trade-Off Between Accuracy and Efficiency

The experimental results reveal a nuanced trade-off mechanism within the proposed WaveHSI framework, manifesting across two dimensions: data scale and decomposition depth.

Firstly, from the perspective of data scale, Table 11 indicates a scale-dependent performance characteristic. On smaller datasets like Indian Pines (145×145×200), the reduction in total processing time is marginal, as the computational overhead of standard decompression is negligible compared to model inference. However, the efficiency advantage becomes increasingly pronounced on the larger Kennedy Space Center dataset (512×614×176), where we achieved the minimum total time. This underscores the core value of compressed-domain computing: as the spatial resolution of sensors scales, the conventional “decode-then-process” paradigm faces severe bottlenecks, whereas our method effectively bypasses the expensive inverse DWT.

Secondly, the multi-resolution nature of wavelets offers an intrinsic flexibility to fine-tune this trade-off by selecting different decoding layers. As analyzed in Table 3, while the full fusion strategy (L3+2+1) yields the highest classification accuracy, intermediate configurations like L3+2 or using the L2 sub-band alone present compelling alternatives for time-sensitive applications. For instance, on the Kennedy Space Center dataset, adopting the L3+2 configuration reduces the decoding time by approximately 76% (173.9 ms → 41.1 ms) compared to L3+2+1, with only a negligible drop in Overall Accuracy (99.50% → 99.25%). This implies that our framework can essentially operate in different modes—ranging from a “high-precision mode” (L3+2+1) to a “high-speed mode” (L3+2)—allowing for adaptive deployment based on the specific latency constraints and computational resources of the target platform (e.g., on-orbit satellites vs. ground stations).

### 7.2. Limitations and Future Work

Despite the significant gains in decoding efficiency and classification accuracy, we acknowledge a limitation regarding pure inference latency. As indicated in the efficiency analysis, our method is computationally heavier during the inference phase compared to lightweight graph-based models (e.g., GraphGST). This latency is primarily largely attributed to the complex architecture of the $M^{2}S^{2}$-FEM module and the quadratic complexity of the stacked cross-attention blocks, which prioritize feature interaction depth over calculation speed. This presents a clear direction for our future research, which will focus on model lightweighting. We intend to explore network pruning and knowledge distillation techniques to streamline the $M^{2}S^{2}$-FEM structure. The objective is to retain the rich feature representation capability of the current model while significantly reducing the parameter count, aiming to achieve a balance where both decoding and inference are optimized for real-time applications.

## 8. Conclusions

In this paper, we pioneered the exploration of HSI classification directly within the compressed domain, offering a feasible scheme to overcome the computational bottleneck of data decompression. By operating on DWT coefficients, the proposed framework bypasses the redundancy of full image reconstruction. Specifically, we designed the $M^{2}S^{2}$-FEM to extract hierarchical spatial–spectral features from wavelet sub-bands, and the SCA-FFM to synergize low-frequency global context with high-frequency textural details. Empirical evidence across three benchmarks demonstrates that our method eliminates at least 56% of the decompression overhead while achieving classification accuracy comparable to state-of-the-art methods that rely on fully decompressed data. We believe that this work establishes a baseline for future research into efficient, end-to-end compressed-domain hyperspectral analysis.

## Figures and Tables

**Table 1:** Comparison of compressed-domain computing methods.

Method	Year	Domain	Task	Strengths	Limitations
*DCT-based Methods*					
Gueguen et al. [4]	2018	DCT	Natural Image Classification	First efficient CNN on JPEG; Significant speedup	Requires heavy CNN modification; Block artifacts
Jeongsoo Park et al. [5]	2023	DCT	Natural Image Classification	ViT structure fits block-features; Fast training	Limited to block-based DCT; No spectral modeling
DCFormer [6]	2023	DCT	Natural Image Cls/Det/Seg	Learnable frequency selection; Effective trade-off	Hard to generalize to hierarchical DWT structures
*DWT-based Methods*					
Chebil et al. [10]	2005	DWT	Natural Image Editing	Efficient for brightness/contrast adjustment	Signal processing only; No semantic feature learning
Zhao et al. [11]	2009	DWT	Watermarking	Effective copyright protection in bitstream	Specific to security tasks; No semantic analysis
Akshara et al. [8]	2020	DWT	RGB Remote Sensing Classification	First DL on compressed RGB remote sensing images	Limited to RGB; Simple architecture lacks fusion
Li et al. [13]	2023	DWT	Whole Slide Image Classification	Efficient pyramid structure for medical slides	Domain specific (Medical); Specific magnification logic
Bisen et al. [12]	2023	DWT	Region Extraction	Segmentation-less text extraction	Specific to document layout; Not for general classification
Ours	-	DWT	HSI Classification	Preserves spectral-spatial info; Cross-attention fusion	-

**Table 2:** Number of training, validation, and testing samples for Indian Pines, Pavia University, and Kennedy Space Center datasets.

No.	Indian Pines				Pavia University				Kennedy Space Center			
	Class	Train.	Val.	Test.	Class	Train.	Val.	Test.	Class	Train.	Val.	Test.
1	Alfalfa	4	1	41	Asphalt	332	33	6266	Scrub	76	8	677
2	Corn-notill	143	14	1271	Meadows	932	93	17,624	Willow-swamp	24	3	216
3	Corn-mintill	83	8	739	Gravel	104	11	1984	Cp-hammock	25	3	228
4	Corn	24	2	211	Trees	154	15	2895	Cp/O-hammock	25	3	224
5	Grass-pasture	48	5	430	Painted-m-s	67	7	1271	Slash-pine	16	2	143
6	Grass-trees	73	7	650	Bare-soil	252	25	4752	Oak/B-hammock	23	2	204
7	Grass-pasture-m	3	1	24	Bitumen	66	7	1257	HW-swamp	11	1	93
8	Hay-windrowed	48	5	425	Self-block-b	184	18	3480	Graminoid-marsh	43	4	384
9	Oats	2	1	17	Shadows	47	5	895	Spartine-marsh	52	5	463
10	Soybean-notill	97	10	865					Cattail-marsh	40	4	360
11	Soybean-mintill	245	25	2185					Salt-marsh	42	4	373
12	Soybean-clean	59	6	528					Mud-flats	50	5	448
13	Wheat	21	2	182					Water	93	9	825
14	Woods	126	13	1126								
15	Buildings-g-t	39	4	343								
16	Stone-steel-t	9	1	83								
Total		1024	105	9120		2138	214	40,424		520	53	4638

**Table 3:** Analysis of Different Decompression Layers. The best results are highlighted in bold.

Dataset		L3	L2	L1	L3+2	L3+2+1	Full Decompression
	Inverse Wavelet Transform	/	L3 → L2	L3 → L2 → L1	L3 → L2	L3 → L2 → L1	L3 → L2 → L1 → FULL
Indian Pines	KA (%)	96.61	97.27	98.27	98.63	**98.68**	/
	OA (%)	96.78	97.43	98.48	98.80	**98.84**	/
	AA (%)	97.03	96.28	98.98	98.08	**99.02**	/
	Decode Time (ms)	/	5.9	14.8	5.9	14.8	33.9
Pavia University	KA (%)	94.39	98.16	99.17	**99.66**	99.63	/
	OA (%)	95.75	98.23	99.25	**99.74**	99.72	/
	AA (%)	94.12	97.90	98.80	**99.39**	99.31	/
	Decode Time (ms)	/	10.4	37.8	10.4	37.8	111.7
Kennedy Space Center	KA (%)	96.59	97.19	99.15	99.19	**99.46**	/
	OA (%)	96.70	97.29	99.22	99.25	**99.50**	/
	AA (%)	96.92	97.36	99.34	99.39	**99.51**	/
	Decode Time (ms)	/	41.1	173.9	41.1	173.9	1305.0

**Table 4:** The impact of λ value on accuracy. The best results are highlighted in bold.

Metric	$1\times 10^{-4}$	$1\times 10^{-3}$	$1\times 10^{-2}$	$1\times 10^{-1}$	1
KA (%)	98.46	98.89	**99.39**	98.89	98.65
OA (%)	98.84	99.16	**99.54**	99.16	98.98
AA (%)	97.73	98.27	**99.07**	98.18	97.92

**Table 5:** Ablation study on SFA loss. The best results are highlighted in bold.

	Metric	L3	L2	L1	L3+2	L3+2+1
	KA (%)	98.67	99.25	99.30	99.13	99.50
Without SFA	OA (%)	98.99	99.44	99.47	99.34	99.63
	AA (%)	97.99	98.84	99.06	98.64	**99.24**
	KA (%)	**98.70**	**99.32**	**99.41**	**99.39**	**99.52**
With SFA	OA (%)	**99.02**	**99.49**	**99.56**	**99.54**	**99.64**
	AA (%)	**98.07**	**99.03**	**99.27**	**99.07**	99.19

**Table 6:** Analysis on the number of cross-attention modules. The best results are highlighted in bold.

	Metric	1	2	3	4	5	6
	KA (%)	97.79	98.14	98.58	98.64	**98.73**	98.49
L3+2	OA (%)	97.94	98.31	98.74	98.79	**98.88**	98.68
	AA (%)	97.52	97.59	98.14	97.66	**98.61**	98.58
	KA (%)	98.44	98.68	98.97	98.76	**99.01**	98.63
L3+2+1	OA (%)	98.63	98.84	99.10	98.92	**99.13**	98.80
	AA (%)	98.24	99.02	98.68	**99.07**	98.38	98.55

**Table 7:** Analysis of different masking strategies. The best results are highlighted in bold.

	Metric	No Mask	All	All	No LL	No LL
			=0.1	=0.01	Others = 0.1	Others = 0.01
	KA (%)	98.63	**98.73**	98.50	98.43	98.40
L3+L2	OA (%)	98.80	**98.88**	98.69	98.62	98.60
	AA (%)	98.08	**98.56**	97.91	97.53	98.33
	KA (%)	98.68	**98.80**	98.26	98.73	98.59
L3+2+1	OA (%)	98.84	**98.95**	98.47	98.88	98.76
	AA (%)	99.02	**99.09**	98.62	99.08	98.39

**Table 8:** Classification Results for the Indian Pines Dataset (%). The 1st, 2nd, and 3rd best results are in bold, underlined, and italic, respectively.

Class	SVM (2004)	RF (2005)	ContextNet (2017)	RSSAN (2020)	SSTN (2022)	SSAN (2020)	SSSAN (2022)	SSAtt (2021)	A2S2KResNet (2021)	CVSSN (2023)	IGroupSS-Mamba (2024)	GraphGST (2024)	Ours
Alfalfa	46.95 ± 13.99	71.04 ± 22.75	95.86 ± 6.64	93.68 ± 11.46	84.29 ± 22.19	97.08 ± 4.52	89.73 ± 9.99	97.10 ± 6.48	95.38 ± 5.56	94.63 ± 5.24	*97.56 ± 1.99*	**99.21 ± 1.12**	99.15 ± 1.21
Corn-notill	75.08 ± 1.92	68.77 ± 1.79	79.48 ± 3.54	79.81 ± 5.57	91.15 ± 4.90	86.33 ± 2.47	93.09 ± 2.85	94.17 ± 1.54	**98.08 ± 0.94**	97.55 ± 0.76	93.07 ± 3.00	*95.64 ± 1.37*	95.25 ± 0.56
Corn-mintill	76.99 ± 4.31	75.93 ± 2.89	82.01 ± 5.14	85.31 ± 4.82	97.78 ± 1.23	85.71 ± 3.27	95.21 ± 1.92	96.92 ± 1.18	98.80 ± 0.90	*99.05 ± 0.86*	**99.78 ± 0.32**	96.16 ± 0.33	99.72 ± 0.31
Corn	63.25 ± 4.34	57.31 ± 5.18	85.20 ± 4.65	79.49 ± 14.25	96.07 ± 3.75	88.87 ± 6.27	92.11 ± 3.79	97.71 ± 1.73	96.19 ± 3.55	*98.57 ± 1.60*	98.59 ± 1.15	93.61 ± 0.96	**100.00 ± 0.00**
Grass-pasture	89.68 ± 3.00	85.67 ± 3.20	94.15 ± 3.13	92.09 ± 4.68	93.02 ± 6.48	93.97 ± 3.57	97.50 ± 1.80	97.26 ± 2.07	98.09 ± 1.74	*98.10 ± 1.45*	98.39 ± 0.99	95.78 ± 0.39	**99.43 ± 0.23**
Grass-trees	89.16 ± 1.55	81.36 ± 1.45	95.88 ± 1.78	94.38 ± 2.22	98.31 ± 1.25	96.08 ± 2.48	98.34 ± 2.38	98.64 ± 1.84	99.48 ± 0.88	*99.62 ± 0.34*	98.22 ± 1.76	**99.95 ± 0.07**	99.68 ± 0.13
Grass-pasture-m	85.52 ± 8.07	32.95 ± 42.12	90.79 ± 11.24	85.43 ± 12.16	83.60 ± 13.08	90.59 ± 11.43	93.40 ± 7.44	97.65 ± 4.71	92.96 ± 10.23	94.33 ± 8.69	**100.00 ± 0.00**	*96.15 ± 0.00*	95.83 ± 3.40
Hay-windrowed	91.34 ± 1.49	85.23 ± 1.72	95.70 ± 2.79	94.73 ± 2.57	98.77 ± 2.50	97.00 ± 2.78	98.08 ± 1.33	98.45 ± 1.13	99.00 ± 1.23	*99.93 ± 0.21*	**100.00 ± 0.00**	100.00 ± 0.00	99.92 ± 0.12
Oats	65.94 ± 13.55	53.33 ± 44.44	91.59 ± 10.96	92.24 ± 10.68	73.47 ± 18.20	86.58 ± 14.01	85.73 ± 10.84	95.96 ± 5.62	**97.09 ± 3.83**	*94.97 ± 10.08*	81.48 ± 18.33	75.93 ± 6.93	92.16 ± 5.54
Soybean-notill	74.10 ± 2.06	72.77 ± 4.40	87.61 ± 3.93	86.52 ± 4.28	92.75 ± 3.58	91.10 ± 3.54	95.56 ± 2.26	94.84 ± 2.34	96.77 ± 1.90	97.53 ± 1.05	*97.52 ± 1.50*	95.93 ± 0.75	**97.66 ± 0.55**
Soybean-mintill	75.63 ± 1.35	69.60 ± 1.47	88.42 ± 2.11	85.57 ± 4.28	95.27 ± 2.12	90.54 ± 1.69	96.18 ± 1.22	95.26 ± 1.57	98.29 ± 0.87	*98.31 ± 0.70*	98.99 ± 0.35	93.82 ± 0.70	**99.11 ± 0.16**
Soybean-clean	78.06 ± 4.72	62.62 ± 5.73	75.12 ± 6.35	73.23 ± 8.45	90.77 ± 6.49	84.54 ± 4.02	89.46 ± 3.36	92.40 ± 2.98	*96.28 ± 2.00*	97.34 ± 1.11	92.63 ± 5.26	90.45 ± 1.00	**98.48 ± 0.52**
Wheat	91.63 ± 5.35	85.38 ± 4.80	97.28 ± 2.93	93.58 ± 5.95	97.62 ± 2.54	96.76 ± 3.48	98.58 ± 2.68	**99.89 ± 0.22**	*99.37 ± 1.28*	99.63 ± 0.79	98.74 ± 0.67	99.10 ± 0.25	99.04 ± 1.35
Woods	91.48 ± 1.23	89.70 ± 0.98	95.68 ± 2.35	95.33 ± 1.30	97.16 ± 1.69	96.02 ± 1.34	97.98 ± 1.08	97.65 ± 0.87	98.89 ± 0.71	98.68 ± 0.64	**99.94 ± 0.08**	*99.50 ± 0.04*	99.87 ± 0.09
Buildings-g-t	78.47 ± 5.12	63.49 ± 5.85	89.88 ± 3.05	86.58 ± 3.82	91.85 ± 3.99	90.27 ± 4.52	93.80 ± 2.23	94.67 ± 1.46	94.62 ± 2.19	95.81 ± 2.07	*96.54 ± 0.71*	**98.95 ± 0.14**	98.78 ± 0.66
Stone-steel-t	*97.44 ± 2.67*	97.12 ± 2.56	90.53 ± 8.17	90.54 ± 9.41	89.30 ± 9.42	92.84 ± 6.14	95.22 ± 5.59	95.28 ± 6.12	96.45 ± 3.11	95.95 ± 3.28	96.03 ± 1.48	**99.21 ± 0.56**	97.47 ± 0.00
KA	77.42 ± 0.59	71.28 ± 0.92	86.32 ± 1.88	84.97 ± 3.98	93.68 ± 2.70	89.59 ± 1.13	94.87 ± 0.69	95.34 ± 0.60	*97.70 ± 0.44*	97.95 ± 0.24	97.30 ± 1.30	95.59 ± 0.36	**98.41 ± 0.19**
OA	80.33 ± 0.50	75.12 ± 0.82	88.04 ± 1.64	86.88 ± 3.45	94.46 ± 2.37	90.89 ± 0.98	95.51 ± 0.61	95.92 ± 0.53	*97.98 ± 0.39*	98.20 ± 0.21	97.63 ± 1.14	96.12 ± 0.31	**98.60 ± 0.17**
AA	79.42 ± 1.69	72.02 ± 3.91	89.70 ± 1.97	88.03 ± 3.34	91.95 ± 3.54	91.52 ± 1.48	94.37 ± 1.23	96.49 ± 0.56	*97.23 ± 1.26*	97.50 ± 1.07	96.72 ± 1.97	95.59 ± 0.66	**98.22 ± 0.74**

**Table 9:** Classification Results for the Pavia University Dataset (%). The 1st, 2nd, and 3rd best results are in bold, underlined, and italic, respectively.

Class	SVM (2004)	RF (2005)	ContextNet (2017)	RSSAN (2020)	SSTN (2022)	SSAN (2020)	SSSAN (2022)	SSAtt (2021)	A2S2KResNet (2021)	CVSSN (2023)	IGroupSS-Mamba (2024)	GraphGST (2024)	Ours
Asphalt	91.23 ± 0.85	89.85 ± 1.34	97.15 ± 0.58	96.51 ± 0.62	97.66 ± 1.04	97.71 ± 0.51	99.05 ± 0.45	97.76 ± 0.58	*99.48 ± 0.16*	99.63 ± 0.19	98.87 ± 1.22	98.65 ± 0.18	**99.94 ± 0.04**
Meadows	92.64 ± 0.35	87.99 ± 0.42	99.15 ± 0.26	99.55 ± 0.29	99.49 ± 0.52	99.66 ± 0.17	99.67 ± 0.16	99.76 ± 0.12	*99.91 ± 0.07*	99.94 ± 0.04	99.79 ± 0.10	99.89 ± 0.07	**99.99 ± 0.00**
Gravel	82.30 ± 1.37	73.81 ± 2.27	92.70 ± 2.41	93.36 ± 2.21	96.84 ± 2.66	95.57 ± 1.40	97.79 ± 1.05	97.61 ± 1.13	*99.25 ± 0.70*	99.43 ± 0.54	97.48 ± 1.88	91.29 ± 1.13	**99.95 ± 0.04**
Trees	96.42 ± 0.68	93.32 ± 1.00	99.12 ± 0.70	99.23 ± 0.39	97.18 ± 1.75	*99.46 ± 0.39*	99.54 ± 0.28	99.29 ± 0.51	**99.64 ± 0.29**	99.33 ± 0.43	99.03 ± 0.29	96.11 ± 1.57	98.55 ± 0.18
Painted-m-s	98.83 ± 0.82	97.16 ± 1.39	*99.76 ± 0.51*	98.76 ± 0.81	99.24 ± 0.73	99.70 ± 0.36	99.70 ± 0.55	99.69 ± 0.28	99.60 ± 0.38	99.82 ± 0.15	99.61 ± 0.17	**100.00 ± 0.00**	99.12 ± 0.37
Bare-soil	94.06 ± 0.70	87.46 ± 1.43	98.67 ± 0.32	98.58 ± 0.51	99.33 ± 1.80	98.94 ± 0.62	99.37 ± 0.36	99.03 ± 0.72	*99.74 ± 0.24*	99.70 ± 0.15	99.36 ± 0.91	99.78 ± 0.02	**100.00 ± 0.00**
Bitumen	85.47 ± 3.02	81.71 ± 2.91	96.15 ± 1.50	96.04 ± 2.02	98.97 ± 1.30	97.31 ± 1.91	99.46 ± 0.57	98.31 ± 1.38	99.63 ± 0.54	*99.65 ± 0.31*	**100.00 ± 0.00**	96.88 ± 0.40	99.83 ± 0.13
Self-block-b	80.59 ± 2.41	77.62 ± 2.11	92.92 ± 1.43	92.27 ± 2.20	96.38 ± 2.10	94.79 ± 1.14	97.12 ± 0.84	95.67 ± 0.97	98.50 ± 0.54	*99.13 ± 0.34*	99.17 ± 0.39	94.86 ± 1.20	**99.51 ± 0.14**
Shadows	**99.94 ± 0.07**	*99.88 ± 0.09*	99.43 ± 0.88	98.17 ± 1.12	96.37 ± 1.48	99.30 ± 0.52	99.57 ± 0.67	99.93 ± 0.11	99.81 ± 0.22	99.51 ± 0.57	97.15 ± 1.39	98.28 ± 0.31	98.08 ± 0.87
KA	88.43 ± 0.42	82.93 ± 0.31	97.15 ± 0.45	97.13 ± 0.62	98.02 ± 0.67	98.08 ± 0.35	98.95 ± 0.22	98.44 ± 0.32	*99.51 ± 0.12*	99.60 ± 0.08	99.09 ± 0.58	97.92 ± 0.09	**99.69 ± 0.07**
OA	91.38 ± 0.31	87.38 ± 0.23	97.85 ± 0.34	97.84 ± 0.46	98.51 ± 0.50	98.55 ± 0.26	99.21 ± 0.17	98.82 ± 0.24	*99.63 ± 0.09*	99.70 ± 0.06	99.32 ± 0.44	98.44 ± 0.07	**99.77 ± 0.05**
AA	91.28 ± 0.42	87.65 ± 0.51	97.23 ± 0.54	96.94 ± 0.68	97.94 ± 0.57	98.05 ± 0.38	99.03 ± 0.20	98.56 ± 0.31	*99.51 ± 0.15*	99.57 ± 0.10	98.94 ± 0.58	97.30 ± 0.20	**99.63 ± 0.27**

**Table 10:** Classification Results for the Kennedy Space Center Dataset (%). The 1st, 2nd, and 3rd best results are in bold, underlined, and italic, respectively.

Class	SVM (2004)	RF (2005)	ContextNet (2017)	RSSAN (2020)	SSTN (2022)	SSAN (2020)	SSSAN (2022)	SSAtt (2021)	A2S2KResNet (2021)	CVSSN (2023)	IGroupSS-Mamba (2024)	GraphGST (2024)	Ours
Scrub	91.16 ± 1.33	91.00 ± 1.38	98.22 ± 1.60	98.09 ± 2.35	99.37 ± 1.48	98.86 ± 1.03	99.21 ± 1.12	99.32 ± 0.87	99.48 ± 0.58	*99.76 ± 0.42*	98.15 ± 1.85	**100.00 ± 0.00**	99.85 ± 0.21
Willow-swamp	89.32 ± 3.72	77.79 ± 5.87	81.89 ± 10.01	93.38 ± 3.88	95.75 ± 3.19	91.51 ± 7.16	96.06 ± 2.37	*95.80 ± 2.41*	**96.88 ± 2.35**	94.27 ± 12.80	78.54 ± 0.37	88.89 ± 6.87	95.17 ± 4.01
Cp-hammock	69.71 ± 5.57	87.72 ± 4.81	77.13 ± 4.58	79.34 ± 6.55	92.39 ± 6.34	77.02 ± 5.09	90.09 ± 5.79	89.41 ± 4.05	87.87 ± 4.52	97.61 ± 1.83	89.28 ± 2.57	*94.53 ± 1.02*	**99.85 ± 0.21**
Cp/O-hammock	49.07 ± 3.26	59.60 ± 4.32	70.78 ± 6.43	72.83 ± 7.56	80.74 ± 7.31	73.63 ± 2.28	82.99 ± 7.81	*84.46 ± 6.04*	86.32 ± 4.13	**90.47 ± 6.49**	73.57 ± 7.18	44.58 ± 8.13	84.23 ± 2.21
Slash-pine	70.71 ± 10.19	70.98 ± 6.46	65.16 ± 5.78	71.08 ± 6.17	82.08 ± 8.37	73.06 ± 7.51	80.52 ± 8.11	84.07 ± 6.02	87.01 ± 5.59	85.10 ± 28.64	*85.75 ± 0.65*	0.22 ± 0.31	**99.77 ± 0.33**
Oak/B-hammock	71.89 ± 7.47	61.87 ± 7.26	85.64 ± 5.36	83.83 ± 8.51	90.58 ± 5.14	85.72 ± 4.37	*91.59 ± 6.40*	90.27 ± 5.75	93.61 ± 5.41	90.81 ± 14.63	89.16 ± 5.02	78.75 ± 19.68	**98.51 ± 1.08**
HW-swamp	75.95 ± 4.12	73.51 ± 3.32	86.99 ± 6.69	90.92 ± 7.31	91.02 ± 5.74	85.01 ± 8.88	89.58 ± 9.94	95.23 ± 6.37	95.22 ± 5.03	*95.92 ± 10.61*	**100.00 ± 0.00**	86.33 ± 7.93	97.10 ± 1.36
Graminoid-marsh	88.36 ± 3.78	76.33 ± 4.62	93.18 ± 2.47	95.85 ± 2.51	96.51 ± 2.30	95.25 ± 4.01	*97.61 ± 2.35*	97.88 ± 1.45	97.50 ± 2.20	**99.61 ± 0.60**	94.24 ± 2.27	88.13 ± 2.09	95.78 ± 2.05
Spartine-marsh	89.17 ± 2.63	84.66 ± 2.57	96.03 ± 2.05	97.73 ± 1.67	98.62 ± 1.18	97.10 ± 2.71	99.20 ± 0.87	99.13 ± 1.35	99.15 ± 0.81	*99.33 ± 1.16*	93.80 ± 3.04	**100.00 ± 0.00**	99.85 ± 0.21
Cattail-marsh	97.22 ± 2.94	89.19 ± 6.49	94.57 ± 2.89	96.69 ± 2.06	98.69 ± 1.78	96.55 ± 2.30	99.39 ± 1.32	99.86 ± 0.23	*99.83 ± 0.28*	**99.97 ± 0.09**	79.49 ± 5.63	98.61 ± 0.75	99.81 ± 0.26
Salt-marsh	96.51 ± 1.19	97.59 ± 1.94	99.45 ± 0.79	99.57 ± 0.58	98.82 ± 2.19	99.56 ± 0.66	**99.95 ± 0.16**	*99.74 ± 0.63*	99.45 ± 0.85	99.95 ± 0.11	99.73 ± 0.38	95.91 ± 0.31	99.73 ± 0.00
Mud-flats	93.37 ± 2.35	92.11 ± 2.71	94.97 ± 3.54	94.45 ± 2.97	98.61 ± 1.69	96.90 ± 2.06	*99.60 ± 0.46*	98.94 ± 0.88	98.38 ± 0.75	99.58 ± 0.61	97.72 ± 1.33	**100.00 ± 0.00**	99.77 ± 0.18
Water	99.80 ± 0.24	98.41 ± 0.63	99.18 ± 1.10	99.06 ± 0.98	99.38 ± 1.05	99.57 ± 0.62	99.81 ± 0.38	99.85 ± 0.36	99.95 ± 0.11	**100.00 ± 0.00**	100.00 ± 0.00	*100.00 ± 0.00*	100.00 ± 0.00
KA	86.87 ± 0.46	84.85 ± 1.10	91.52 ± 1.49	93.04 ± 1.33	95.84 ± 0.99	93.24 ± 1.52	96.32 ± 0.92	96.61 ± 0.55	*96.85 ± 0.50*	97.64 ± 2.88	92.29 ± 0.96	89.76 ± 1.74	**98.25 ± 0.33**
OA	88.22 ± 0.42	86.41 ± 0.99	92.39 ± 1.34	93.75 ± 1.19	96.26 ± 0.89	93.93 ± 1.36	96.70 ± 0.83	96.95 ± 0.50	*97.17 ± 0.45*	97.88 ± 2.58	93.08 ± 0.86	90.82 ± 1.56	**98.43 ± 0.29**
AA	83.25 ± 1.25	81.60 ± 1.55	87.94 ± 2.17	90.22 ± 2.06	94.04 ± 1.14	89.98 ± 2.33	94.28 ± 1.70	94.92 ± 1.06	*95.43 ± 0.87*	96.34 ± 4.96	90.72 ± 0.80	82.77 ± 2.15	**97.65 ± 0.48**

**Table 11:** Comparison of computational efficiency on three datasets (Unit: milliseconds). The total time is the sum of decode time and inference time. The best results are highlighted in bold.

Dataset	Metric	IGroupSS-Mamba	GraphGST	Ours
		(2024)	(2024)	(L3+2+1)
Indian Pines (145×145×200)	Decode time	33.9	33.9	**14.8**
	Inference time	1475.7	**156.4**	433.3
	Total time	1509.6	**190.3**	448.1
Pavia University (610×340×103)	Decode time	111.7	111.7	**37.8**
	Inference time	7330.1	**475.3**	1384.7
	Total time	7441.8	**587.0**	1422.5
Kennedy Space Center (512×614×176)	Decode time	1305.0	1305.0	**173.9**
	Inference time	1888.5	**78.6**	342.7
	Total time	3193.5	1383.6	**516.6**

## Data Availability

The data presented in this study are openly available in [Hyperspectral Remote Sensing Scenes] at [https://www.ehu.eus/ccwintco/index.php/Hyperspectral_Remote_Sensing_Scenes (accessed on 6 January 2025).

## Abbreviations

The following abbreviations are used in this manuscript:

HSI	Hyperspectral Image
DCT	Discrete Cosine Transform
DWT	Discrete Wavelet Transform
EBCOT	Embedded Block Coding with Optimized Truncation
JPEG	Joint Photographic Experts Group
JPEG2000	Joint Photographic Experts Group 2000
LL	low-frequency sub-band
HL, LH, HH	high-frequency sub-bands
MOE	Mixture of Experts
$M^{2}S^{2}$-FEM	Multi-branch Multi-scale Spatial–Spectral Feature Extraction Module
SCA-FFM	Sub-band Cross-attention Feature Fusion
SFA	Sub-band Feature Alignment
KA	Kappa coefficient
OA	Overall Accuracy
AA	Average Accuracy
SE	Squeeze-and-Excitation
RF	Random Forest
SVM	Support Vector Machine
L1, L2, L3	1st, 2nd, and 3rd Wavelet Decomposition Levels
L3+2	Combination of L3 and L2
L3+2+1	Combination of L3, L2 and L1
FULL	full decompression
